# Amino acids and protein profiles of defatted camel and cow milk fractions: correlation with their *in vitro* antioxidant and antidiabetic activities

**DOI:** 10.3389/fnut.2023.1295878

**Published:** 2024-01-10

**Authors:** Nouha Harizi, Ahmed Zouari, Nesrine Rokbeni, Malek Ben Zid, Nouha M’hiri, Ali Salem, Mohamed Ali Ayadi, Nourhene Boudhrioua

**Affiliations:** ^1^Laboratory of Physiopathology, Food and Biomolecules, LR17ES03, Higher Institute of Biotechnology of Sidi Thabet, University of Manouba, Ariana, Tunisia; ^2^Laboratory of Analyses, Valorization and Food Safety, Food Engineering School of Sfax, University of Sfax, Sfax, Tunisia; ^3^Biological Engineering Department, University Institute of Technology of Saint-Brieuc (IUT Saint-Brieuc), University of Rennes, Saint-Brieuc, France; ^4^Laboratory of Enzyme Engineering and Microbiology, Engineering National School of Sfax (ENIS), University of Sfax, Sfax, Tunisia; ^5^High Institute of Applied Biology of Medenine, University of Gabes, Medinine, Tunisia; ^6^Laboratory of Quality and Safety of Agro-food Products, Gembloux Agro-Bio Tech, University of Liège, Gembloux, Belgium

**Keywords:** camel milk, cow milk, protein fraction, amino acids, antioxidant activity, α-amylase inhibition

## Abstract

**Introduction:**

This work presents proteins, amino acids profiles and antioxidant and properties of camel and cow milk fractions produced using an integrated coagulation-centrifugation process.

**Methods:**

Antioxidant activity using DPPH radical scavenging assay; and antidiabetic activity antidiabetic activity using *in vitro* α-amylase inhibitory activity were assessed on defatted milk fractions and their extracts using water/ethanol or HCl/ethanol solvents. Protein profiles and amino acids composition were analyzed by high-performance liquid chromatography.

**Results and discussions:**

The predominant protein found in cow and camel milk was β-casein in sodium caseinate, β-lactoglobulin was found in the whey of cow milk, whereas α-lactalbumin was detected in the whey fractions of camel. The primary amino acids (comprising 1% to 5.2%) in skim milk and sweet whey milk were leucine, proline, and lysine. However, acid whey, casein fractions (sodium caseinate, and β-casein) from both camel and cow milk exhibited elevated concentrations of histidine, leucine, lysine and proline (1.12 - 6.62%). Camel milk and its different protein fractions showed an interesting *in vitro* α-amylase inhibitory activity varying, according to different milk fractions and extraction methods, from 19.10 ± 1.40 to 97.40 ± 1.50%. Whatever the used method, the whey fractions from camel milk, both acid and sweet, displayed ed the highest antioxidant activity. Principal components analysis showed a positive correlation between the total phenols content, antioxidant (DPPH assay) and antidiabetic (α amylase inhibition test) activities within the milk fractions. Sweet and acid cow milk fractions seem to be the most promising for deeper exploration of *in vivo* biological activities and are promising milk derivatives for specific nutritional diet and/or functional food formulation.

## Graphical Abstract

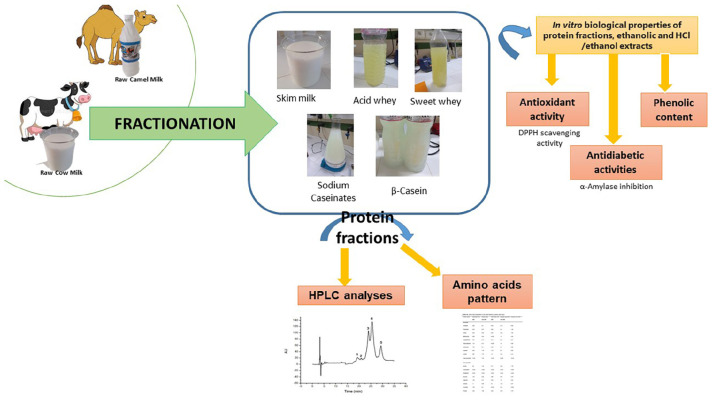

## 1 Introduction

Milk is a valuable diet because of its well-balanced mix of several nutrients and micro-elements (proteins, lactose, fats, vitamins and minerals) (1). Cow milk remains worldwide, the predominant global milk source, maintaining a pivotal role in the international financial system, accounting for about 734 million tons in 2020 (2). The production of camel milk is actively expanding in numerous semi-arid and desert locations, particularly across Africa and Asia due to the increased demand. As per the FAO, global camel population statistics indicate the presence of approximately 40 million Bactrian and dromedary camels, with worldwide camel milk production reaching approximately 3,114 million tons in 2021 (3). Since ancient times, camel milk has been the primary food source for nomadic peoples, who often ingest it fresh or fermented. In the majority of these pastoral Saharan areas, it is regarded as the main food source for a considerable part of the year. Camel milk proteins contain 47% more β-casein than cow milk proteins and 3.5% less κ-casein (4). Additionally, the profile of whey proteins in cow milk revealed that β-lactoglobulin (β-Lg) and α-lactalbumin (α-La) predominated (5). The absence of β-Lg, the over-expression of α -La, and the inclusion of distinctive proteins like peptidoglycan recognition protein (PGRP), make camel milk whey unique (6). In terms of human nutrition, casein and whey protein amino acid compositions hold a special place. These proteins are categorized as having a high nutritional quality, high digestibility (ranging from 97 to 98%), and quick assimilation and usage by the organism. Because they deliver amino acids slowly and continuously into the bloodstream, caseins in particular are an effective source of nutrients. For maintaining tissue growth, healing, and avoiding catabolic processes during exercise (7). Due to its high immunoglobulin and insulin concentrations, camel milk is distinct from other ruminant milk on the basis of both composition and usefulness. This milk gives consumers all the necessary micro-nutrients, including the fatty acids omega-3, conjugated linoleic acid, and oleic acid, that are already present in cow milk, as well as bioactive substances like antioxidants (8). Camel milk, known for its richness in vitamin C, exhibits antioxidant properties attributed to sulfur-containing amino acids, carotenoids, vitamins A and E, as well as enzymes like catalase, superoxide dismutase, and glutathione peroxidase (9, 10). Equol, a phenolic compound originating from daidzein with its scientifically validated antioxidant properties, is also present in milk in large proportions (11). It has been reported that people with type 1 diabetes and consuming raw camel milk, may need to use 30–35% less insulin each day (12). Camel milk is used to treat diabetes as well as to lower blood sugar, minimize insulin resistance, and enhance lipid profiles (13). In addition, Kumar et al. (14) show that camel milk has anti-diabetic properties against both type 1 and type 2 diabetes because it contains modest levels of immunoglobulins, insulin, and insulin-like compounds (13). On the other hand, some authors have shown that whole camel milk and fermented milk has antidiabetic properties via suppressing α-amylase and/or α-glucosidase activities as well as its antioxidant properties (15, 16). However, none of the earlier investigations studied the variations of antioxidant and anti-diabetic properties of different rich protein fractions obtained through fractionation process from camel and cow milk and their correlation with total phenols, proteins and amino-acids profiles. Indeed, fractionating camel milk and cow milk into various protein fractions and characterization of their anti-oxidant and biological properties could be promising for future industrial dairy applications to serve consumers needing specific diets and/or to promote valorization of whey protein which is the most by-product of cheese and butter manufacturing. Thus, the purpose of this research is to present an integrated easy-to-use fractionation approach of camel milk and cow milk along with a detailed analysis of their protein and amino-acids profiles, as well as an *in vitro* assessment of the antioxidant and anti-diabetic characteristics of different milk fractions in order to promote their promising applications as nutraceuticals or food supplements. In this study, cow milk is used as a control because of its prominent status as one of the most widely consumed and culturally ingrained types of milk globally. The consistently well-documented nutritional composition of cow milk serves to substantiate its role as a control. Its historical and widespread usage makes it an ideal reference for standardization purposes in the dairy research.

## 2 Materials and methods

### 2.1 Milk fractions preparation

#### 2.1.1 Sample collection for analysis

Camel (*Camelus dromedarius*) and cow (*Bos taurus*) milk were aseptically collected from 10 to 15 milking females (2–10 months in lactation stage) located on extensive breeding dairy farms in the south of Tunisia. Each milk sample represents a mixture of collected milk from multiple individuals, ensuring a well-rounded blend of milk from the respective dromedaries or cows. This methodology allows providing a comprehensive and representative sampling of the milk samples used in the experiments as reported by Zouari et al. (4). Milk samples were delivered to the laboratory et 4°C. Samples were frozen 24 to 72 h at −20°C until use. The defrosting of milk samples took 2 h at 4°C. Before beginning any additional treatment, the pH is routinely checked. After the thawing process, skimming was executed in the following manner: cow milk underwent a single centrifugation step at 2000 × *g* for 15 min at 5°C, whereas camel milk underwent three consecutive centrifugation steps at 2000 × *g* for 15 min at 5°C (4, 5).

#### 2.1.2 Casein and whey fraction separation

The acid whey fraction was obtained by precipitating the whey from the casein at a pH of 4.3 for camel milk and 4.6 for cow milk, adding HCl (12 N), and centrifuging at 5000 × *g* for 15 min. By dissolving with NaOH (1 M), the sodium caseinate fraction is produced. To produce the sweet whey fraction, enzymatic coagulation was conducted with rennet enzymes (Parachimic, Laboratories Arrazi, Sfax, Tunisia, strength = 1: 10,000) at a temperature of 37°C. Following the separation of the whey and casein fractions by rennet, an amount of desalinated water was incorporated to the curd. To inhibit rennet activity, the entire content was then placed in an 80°C boiling water bath for 5 min. It was then kept at 4°C for up to 24 h before separating the β-casein by centrifugation at 5000 × *g* for 15 min at 5°C (17). In this study, cow milk is chosen as a control for standardization purposes due to its historical and widespread usage. All analyses were performed in triplicate for both types of milk.

### 2.2 Protein profiles analyses

The chromatographic analyses were conducted employing an Agilent-1100 RP-HPLC equipment (Agilent Technologies, Waldbronn, Germany) following the Yüksel and Erdem method (18) as well as the procedure outlined by Jafar et al. (19). To separate milk proteins, a C18 column RP-HPLC (Zorbax Eclipse Plus C18, batch number: B14292) was employed with the following specifications: 250 mm in length, 4.6 mm in diameter, and a particle size of 5 mm. Analysis was then conducted with a Shimadzu SPD6A UV detector for a duration of 40 min at 220 nm. A volume of 500 mL of milk sample was combined in a 70:30 (volume/volume) ratio with a 3.7 mL solution of solvent A made with a mixture of acetonitrile, water, and trifluoroacetic acid in a volume ratio of 100:900:1 and solvent B made with a mixture of acetonitrile, water, and trifluoroacetic acid in a volume ratio of 900:100:1. The sample underwent filtering through a 0.45 m filter before being injected into the column in a 20 μL volume. Following sample injection, a gradient was created by progressively raising the amount of solvent B over a period of time, beginning at 20% and progressing to 46% by reaching the end of the run. Individual standard proteins were separately diluted in a 70:30 (volume/volume) combination of solvents A and B.

### 2.3 Amino acids analysis

Amino acids were quantified according to Maria and Federico (20). The procedure involved hydrolyzing the sample with a 6N HCl solution, followed by the extraction of total amino acids from freeze-dried milk fractions. Specifically, 1 mL of 6N HCl was introduced into a screw tube containing 40 mg of freeze-dried sample and incubated at 110°C for 24 h. The created acidic solution was neutralized by adding an equivalent amount of NaOH (6N). Subsequently, the mixture was then filtered through a 0.2 m syringe filter and kept at 4°C.

For analysis, 100 μL of the filtered sample solution was sequentially combined with 500 μL of borate buffer, 100 μL of O-phthalaldehyde, and 100 μL of FMOC (9-fluorenyl methyl chloroformate). After filtering via a 0.2 μm filter, the mixture was submitted to HPLC (high-performance liquid chromatography) on a Zorbax Eclipse AAA column. At a flow rate of 1.5 mL/min, a temperature of 40°C, and an injection volume of 20 μL, the HPLC analysis was performed. Principal amino acids were identified at 338 nm, whereas secondary amino acids, proline and hydroxyproline, were discovered at 262 nm. To estimate the amount of each amino acid, standard ranges were established using a combination of amino acids.

### 2.4 Analysis of phenolic compounds and antioxidant capacity

The assessment of DPPH radical scavenging capacity was conducted directly on liquid milk fractions and involved two distinct extraction methods. The first method followed the procedure outlined by Li et al. (21) and employed a conventional solution containing HCl (1N) / 95% ethanol (v/v, 15/85). In the second extraction method, a combination of deionized water and ethanol (15/85 v/v) was used. In both methods, 20 mL of the respective solvent was added to 3.0 g of milk placed in brown 50-mL bottles. The bottles were then shaken for 1 h at 30°C. Subsequently, the solvent-milk mixture was subjected to centrifugation at 5°C for 15 min at 7800 × *g* (SS -34 Rotors on an RC5C Sorvall Instruments, DuPont, Wilmington, DE, USA). The resulting supernatant liquids were stored at 20°C in darkness until the DPPH scavenging activity and total phenolic content (TPC) were determined.

Total phenolic content was determined in the extracts using the procedure reported by Singleton et al. (22). The extracts were combined with Folin-Ciocalteu reagent and Na_2_CO_3_ solution, and the mixture was kept in a basin of water at 40°C for 30 min before spectrophotometric analysis. The total phenolic content was quantified at 765 nm and represented as Gallic Acid Equivalent (GAE) per liter of milk.

The radical scavenging activity (RSA) of samples was determined using the DPPH test (23) with minor modifications. A DPPH solution with a concentration of 63.4 M of 1,1-diphenyl-2-picrylhydrazyl (DPPH) was produced by mixing 10 mg of DPPH in 10 mL of methanol. Subsequently, 400 μL of the sample extract was combined with 2.4 mL of the DPPH solution and allowed to incubate for 30 min in darkness. The decrease of DPPH radicals was evaluated through determining the absorbance at 517 nm. The radical scavenging activity was estimated employing the corresponding formula, where A_0_ indicates the starting optical density, and A_*t*_ is the finished optical density.

$$\text{RS}A\left(\%\right)=\frac{\left({\text{A}}_{0}-{\text{A}}_{t}\right)}{{\text{A}}_{0}}$$

### 2.5 Antidiabetic activity

The evaluation of antidiabetic activity involved the assessment of *in vitro* α-amylase inhibitory activity on liquid milk fractions, as well as on extracts prepared using the methods mentioned earlier for DPPH-RSA (HCl (1N) / 95% ethanol (v/v, 15/85) and water/ethanol (15/85 v/v) extracts). In a 1.5 mL centrifuge tube, 20.0 μL of the sample solution and 20.0 μL of α-amylase solution (5.0 U/mL) were combined and maintained at 37°C for 10 min. Subsequently, 40.0 μL of starch solution (0.5%, w/v) was incorporated and reacted for another 10 min at 37°C. The mixture was then incubated in boiling water for 5 min with 80.0 μL of 3,5-dinitrosalicylic acid (DNS) solution. The absorbance was determined at 540 nm (24).

$$\text{I}nhibition,\text{I}\alpha \left(\%\right)=\frac{\left({\text{A}}_{0}-{\text{A}}_{E}\right)}{{\text{A}}_{0}}$$

where A_0_ represents the absorbance of the blank and A_*E*_, according to the equation represents the absorbance of the extract.

### 2.6 Statistical analysis

All experiments were conducted in triplicate, and the mean data were employed for statistical evaluation. The statistical variations were assessed using the XLSTAT 18 program. A one-way analysis of variance (ANOVA) of measures was employed for comparing the samples, followed by Tukey’s test, with significance set at *p*-value < 0.05. The metrics that showed significant variations were subjected to principal component analysis (PCA), including total phenol content, RSA-DPPH, total essential amino acids (TEAA), and α-amylase inhibition percentage, Iα. The PCA was performed with a two-dimensional configuration to ensure clarity in interpretation and reproducibility.

## 3 Results

### 3.1 HPLC profiles of milk protein fractions

**FIGURE 1:**
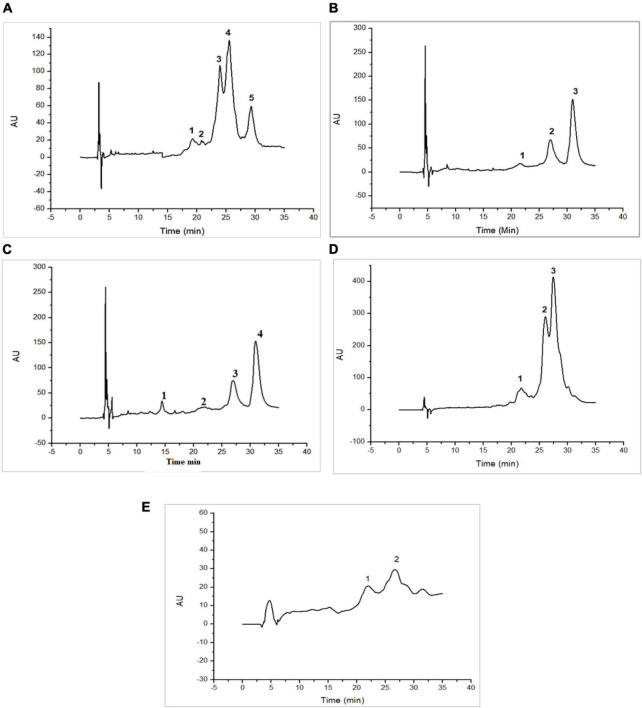
High-performance liquid chromatography (HPLC) chromatograms recorded at 220 nm for native (20°C) cow milk fractions: skim cow milk **(A)**; acid whey fraction **(B)**; sweet whey fraction **(C)**; sodium caseinate fraction **(D)** and β-casein fraction **(E)** AU, arbitrary unit; min, minutes.

**FIGURE 2:**
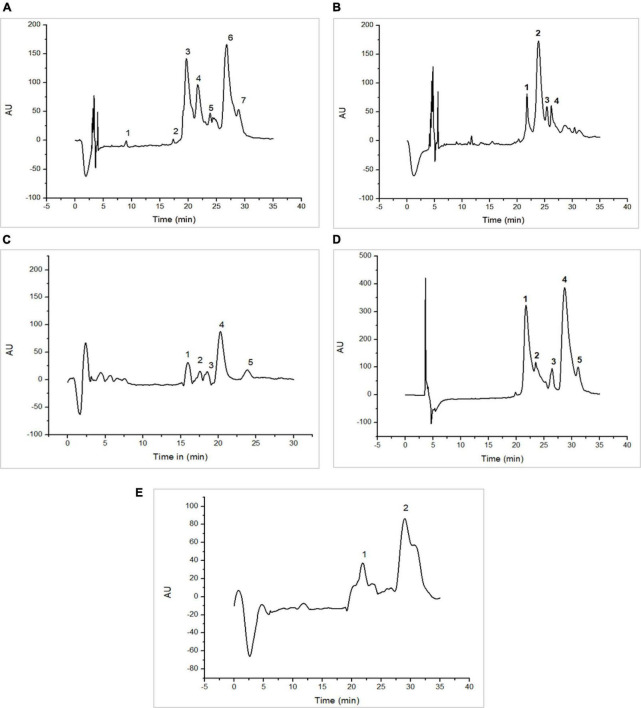
High-performance liquid chromatography (HPLC) chromatograms recorded at 220 nm for native (20°C) camel milk fractions. skim milk **(A)**; acid whey fraction **(B)**; sweet whey fraction **(C)**; sodium caseinate fraction **(D)** and β-casein fraction **(E)** AU, arbitrary unit; min, minutes.

Figures 1, 2 as well as Tables 1, 2, show the protein content of camel and cow milk, including their respective fractions. The HPLC chromatograms for skim milk protein fractions from cows (Figure 1) and camel (Figure 2) revealed notable distinctions. In skim cow milk, four prominent peaks were observed at retention times (RT) of 19.65, 24.61, 26.47, and 29.87 min, representing κ-casein (7.6%), β-casein (43.82%), and β-Lactoglobulin (14.29%). In the chromatograms for skim camel milk (Figure 2), five distinct protein peaks with retention times approximately at 17.50, 20.01, 21.83, 24.27, and 26.93 min were detected. Among these, the primary proteins were those with RT values of 20.01 and 26.93 min, representing αs1-casein (25.60%) and β-casein (34.03%), correspondingly.

**TABLE 1:** The protein identification of cow milk fractions.

Fractions	Number	Identification	RT (min)	Percentage (%)
**Skim milk**				
	1	κ-casein	∼19.65	7.64
	2	n.id	–	3.23
	3	α-casein	∼24.61	31.01
	4	β-casein	∼26.47	43.82
	5	β-lactoglobulin	∼29.87	14.29
**Acid whey**				
	1	BSA	∼21.38	11.38
	2	α-Lactalbumin	∼27.31	34.13
	3	β-lactoglobulin	∼31.05	54.39
**Sweet whey**				
	1	Casein-macropeptides	∼14	6.71
	2	BSA	∼21.75	14.56
	3	α-lactalbumin	∼27.2	29.49
	4	β-lactoglobulin	∼31.25	49.24
**Sodium caseinate**				
	1	κ-casein	∼22	8.55
	2	α-casein	∼26.25	33.24
	3	β-casein	∼27.5	58.20
β **-casein**				
	1	Impurity (mixture of caseins and serum proteins)	∼22	33.16
	2	β-casein	∼27	66.84

RT, retention time; min, minutes; n.id, non-identified; BSA, bovine serum albumin; (-), not determined.

**TABLE 2:** The protein identification of camel milk fractions.

Fractions	Number	Identification	RT (min)	Percentage (%)
**Skim milk**				
	1	n.id	–	1.04
	2	κ-casein	∼17.50	1.96
	3	αs1-casein	∼20.01	25.6
	4	α-Lactalbumin	∼21.83	18.1
	5	CSA	∼24.27	18.1
	6	β-casein	∼26.93	34.03
	7	n.id		8.5
**Acid whey**				
	1	Fragment of α-lactalbumine	∼22.05	16.61
	2	α-Lactalbumin	∼23.35	61.46
	3	CSA	∼25.8	9.67
	4	PGRP	∼26.1	12.25
**Sweet whey**				
	1	n.id	–	17.16
	2	n.id	–	13.63
	3	n.id	–	9.37
	4	α-Lactalbumin	∼20.1	48.96
	5	PGRP	∼23.75	9.45
**Sodium caseinate**				
	1	αs1-casein	∼22	28.16
	2	αs2-casein	∼23.75	13.39
	3	n.id	–	6.34
	4	β-casein	∼28.75	44.56
	5	n.id	∼33.75	7.55
β **-casein**				
	1	Impurity (mixture of caseins and serum proteins)	∼22	32.14
	2	β-casein	∼29	67.86

RT, retention time; min, minutes; CSA, camel serum albumin; n.id, non-identified; PGRP, Peptido-glycan recognition Protein; (-), not determined.

In the chromatograms of cow acid whey (Figure 1), three prominent protein peaks were identified with retention times (RT) approximately at 21.38, 27.31, and 31.05 min. These peaks were identified and characterized as cow serum albumin (11.38%), α-lactalbumin (34.13%), and β-lactoglobulin (54.39%), respectively. In the sweet whey fractions, cow milk exhibited similar primary peaks to those found in acid whey, which included bovine serum albumin (BSA), α-lactalbumin, and β-lactoglobulin. For the cow milk fraction, the predominant proteins were β-lactoglobulin and α-lactalbumin, while in the sweet whey of camel milk, the primary proteins were α-lactalbumin and PGRP. Specifically, α-lactalbumin held the top position as the dominant protein in camel milk whey, while β-lactoglobulin took precedence in the cow milk whey fractions.

Three major protein peaks were identified in HPLC chromatograms of sodium caseinate fraction of cow milk (Figure 1). The proteins were characterized as follows: κ-casein (8.55% of the entire milk proteins), α-casein (33.24% of the entire milk proteins) and β-casein (58.20% of the entire milk proteins). Sodium caseinate of camel milk (Figure 2) contained a high concentration of β-casein, indicating its prominent role as the primary protein within the sodium caseinate camel milk fraction, accounting for 44.56% of its composition. The milk fractionating protocol allows to obtain rich β-casein fractions accounting for 66.84% in cow milk and 67.86% in camel milk.

### 3.2 Amino acid profile of protein fractions in milk

Tables 3, 4 provide insights into the amino acid content of various fractions of camel and cow milk. These analyses reveal an adequate equilibrium of amino acids, characterized by distinct profiles and concentrations of different types of amino acids. Leucine emerged as the predominant amino acid in both skim milk and sweet whey of cow and camel milk protein fractions, while lysine held the second position among essential amino acids across these fractions. Notably, the acid whey and β-casein camel milk fractions presented the highest histidine content (6.38 ± 0.01 g amino acid/100 g protein and 7.3 ± 0.01 g amino acid/100 g protein, respectively) if compared to the cow milk fractions (6.29 ± 0.01 g amino acid/100 g protein and 6.62 ± 0.01 g amino acid/100 g protein, respectively). The current study identified a significant amount of essential amino acids such as threonine, valine, phenylalanine and isoleucine in the sodium caseinate fractions. Camel β-casein fraction had the highest concentration of valine (3.85 ± 0.02 g amino acid/100 g protein) compared to cow milk β-casein fraction (1.63 ± 0.02 g amino acid/100 g protein). Comparatively, camel milk fractions displayed a higher total content of essential amino acids in contrast to cow milk fractions, especially in the case of sodium caseinate (29.21 vs. 20.99 g amino acid/100 g) and β-casein (30.39 vs. 16.85 g amino acid/100 g). These specific fractions, sodium caseinate and β-casein, proved to be the richest in essential amino acids and presented intriguing amino acid profiles. Notably, sodium caseinate exceeded 1.4/100 g of methionine. Furthermore, all milk fractions exhibited concentrations of leucine, lysine, and proline exceeding 1/100 g.

**TABLE 3:** Amino acid composition of freeze-dried cow milk fractions (g amino acid/100 g freeze dried fraction), *n* = 3.

Amino acids	Skim milk	Sweet whey	Acid whey	Sodium caseinate	β -casein
Histidine	2.65 ± 0.01^a^	0.2 ± 0.01^a^	6.29 ± 0.01^a^	3.12 ± 0.01^a^	6.62 ± 0.01^a^
Threonine	2.08 ± 0.03^c^	0.87 ± 0.03^f^	0.92 ± 0.03^f^	2.4 ± 0.03^b^	1.25 ± 0.03^e^
Valine	2.09 ± 0.02^d^	0.84 ± 0.02^f^	0.54 ± 0.02^h^	3.45 ± 0.02^b^	1.63 ± 0.02^e^
Methionine	0.56 ± 0.01^e^	0.23 ± 0.01^g^	0.03 ± 0.01^i^	1.4 ± 0.01^c^	0.32 ± 0.01^f^
Tryptophane	0.47 ± 0.08^a^	0.14 ± 0.08^c^^,^^d^	0.13 ± 0.08^c^^,^^d^	0.31 ± 0.08^a^^,^^b^^,^^c^	0.37 ± 0.08^a^^,^^b^
Phenylalanine	1.33 ± 0.01^c^	0.41 ± 0.01^e^	0.049 ± 0.01^f^	1.8 ± 0.01^c^	0.92 ± 0.01^d^
Isoleucine	1.63 ± 0.01^a^	0.8 ± 0.01^a^	0.36 ± 0.01^a^	2.7 ± 0.01^a^	0.98 ± 0.01^a^
Leucine	3.64 ± 0.03^c^	1.78 ± 0.03^f^	1.12 ± 0.03^h^	2.3 ± 0.03^e^	2.63 ± 0.03^d^
Lysine	3.97 ± 0.08^b^	1.75 ± 0.08^e^	1.2 ± 0.08^f^	3.51 ± 0.08^c^	2.13 ± 0.08^d^
Total	18.42	7.02	10.64	20.99	16.85
Serine	2.5 ± 0.02^d^	1.04 ± 0.02^f^	0.71 ± 0.02^h^	0.64 ± 0.02^i^	1.79 ± 0.02e
Asparagine	0.025 ± 0.01^a^	0.025 ± 0.01^a^	0.025 ± 0.01^a^	0.025 ± 0.01^a^	0.025 ± 0.01^a^
Glutamine	0.034 ± 0.01^a^	0.034 ± 0.01^a^	0.034 ± 0.01^a^	0.034 ± 0.01^a^	0.034 ± 0.01^a^
Glycine	1.02 ± 0.01^a^	0.25 ± 0.01^a^	0.66 ± 0.01^a^	0.67 ± 0.01^a^	0.79 ± 0.01^a^
Arginine	1.98 ± 0.01^b^	0.41 ± 0.01^h^	0.09 ± 0.01^j^	1.9 ± 0.01^c^	0.54 ± 0.01^g^
Alanine	1.1 ± 0.06^a^	0.58 ± 0.06^a^	0.4 ± 0.06^a^	1.24 ± 0.06^a^	0.37 ± 0.06^a^
Tyrosine	1.09 ± 0.01^b^	0.2 ± 0.01^d^	0.033 ± 0.01^e^	2.41 ± 0.01^a^	0.033 ± 0.01^e^
Proline	4.26 ± 0.1^d^	1.89 ± 0.1^h^	2.82 ± 0.1^f^	4.25 ± 0.1^d^	3.79 ± 0.1^a^
Total	12.01	4.43	4.77	11.17	7.37

In each column, different letters mean significant differences between average values (*p* < 0.05).

**TABLE 4:** Amino acid profile of freeze-dried camel milk fractions (g amino acid/100 g of freeze-dried fraction), *n* = 3.

Amino acids	Skim milk	Sweet whey	Acid whey	Sodium caseinate	β -casein
Histidine	1.04 ± 0.01^a^	0.24 ± 0.01^a^	6.38 ± 0.01^a^	1.84 ± 0.01^a^	7.3 ± 0.01^a^
Threonine	1.88 ± 0.03^d^	0.5 ± 0.03^g^	1.17 ± 0.03^e^	2.98 ± 0.03^a^	2.91 ± 0.03^a^
Valine	2.21 ± 0.02^c^	0.57 ± 0.02^g^^,^^h^	0.6 ± 0.02^g^	3.41 ± 0.02^b^	3.85 ± 0.02^a^
Methionine	0.99 ± 0.01^d^	0.11 ± 0.01^h^	0.03 ± 0.01^i^	1.77 ± 0.01^b^	1.79 ± 0.01^a^
Tryptophane	0.33 ± 0.08^a^^,^^b^^,^^c^	0.05 ± 0.08^d^	0.16 ± 0.08^b^^,^^c^^,^^d^	0.49 ± 0.08^a^	0.37 ± 0.08^a^^,^^b^
Phenylalanine	1.61 ± 0.01^c^	0.26 ± 0.01^e^^,^^f^	0.15 ± 0.01^e^^,^^f^	2.7 ± 0.01^a^	1.99 ± 0.01^b^
Isoleucine	2.13 ± 0.01^a^	0.42 ± 0.01^a^	0.38 ± 0.01^a^	3.49 ± 0.01^a^	2.65 ± 0.01^a^
Leucine	3.99 ± 0.03^b^	1.04 ± 0.03^h^	1.23 ± 0.03^g^	6.45 ± 0.03^a^	6.38 ± 0.03^a^
Lysine	3.86 ± 0.08^b^	1.26 ± 0.08^f^	1.6 ± 0.08^e^	6.08 ± 0.08^a^	3.48 ± 0.08^c^
Total	18.04	4.45	11.7	29.21	30.39
Serine	2.62 ± 0.02^c^	0.92 ± 0.02^g^	1.02 ± 0.02^f^	3.85 ± 0.02^a^	2.94 ± 0.02^b^
Asparagine	0.025 ± 0.01^a^	0.025 ± 0.01^a^	0.025 ± 0.01^a^	0.025 ± 0.01^a^	0.025 ± 0.01^a^
Glutamine	0.034 ± 0.01^a^	0.034 ± 0.01^a^	0.034 ± 0.01^a^	0.034 ± 0.01^a^	0.034 ± 0.01^a^
Glycine	0.47 ± 0.01^a^	0.25 ± 0.01^a^	0.81 ± 0.01^a^	0.63 ± 0.01^a^	0.7 ± 0.01^a^
Arginine	1.73 ± 0.01^d^	0.37 ± 0.01^i^	0.57 ± 0.01^f^	2.84 ± 0.01^a^	1.27 ± 0.01^e^
Alanine	0.88 ± 0.06^a^	0.35 ± 0.06^a^	0.3 ± 0.06^a^	1.28 ± 0.06^a^	0.78 ± 0.06^a^
Tyrosine	1.1 ± 0.01^b^	0.033 ± 0.01^e^	0.033 ± 0.01^e^	2.41 ± 0.01^a^	0.92 ± 0.01^c^
Proline	5.17 ± 0.1^c^	1.3 ± 0.1^i^	2.31 ± 0.1^g^	6.03 ± 0.1^b^	7.99 ± 0.1^a^
Total	12.03	3.28	5.1	17.1	14.66

In each column, different letters mean significant differences between average values (*p* < 0.05).

### 3.3 Phenolic compound levels and antioxidant potential

The data for total phenolic content (TPC) and the assessment of antioxidant activity using the DPPH assay for cow and camel skim milk, along with their respective whey and casein fractions, are presented in Table 5. The results reveal significant variability in TPC levels across different milk fractions within milk species (skim milk, whey and casein fractions). Additionally, TPC levels vary depending on the extraction solvent used, either HCl/ethanol or water/ethanol. Notably, the milk fractionation process appears to lead to a reduction in total phenolic content (*p* < 0.05) in HCl/ethanol extracts. TPC content of HCl/ethanol extracts differ significantly between cow and camel milk fractions, with higher levels for camel milk and skim camel milk (46 ± 6.4 mg GAE /L and 40.83 ± 8.5 mg GAE /L, respectively). However, TPC is significantly lower (*p* < 0.05) in camel milk whey fractions (SW CaM, and AW CaM) when compared to camel milk (CaM). Based on the findings, it is possible to deduce that acid extraction of milk results in milk fractions with lower TPC content than the equivalent skim milk. On the other hand, water/ethanol extracts show higher TPC values compared to HCl/ethanol extracts, with notable variability among fractions. For instance, the ethanolic extracts of camel milk casein fractions exhibit higher TPC levels than those of the acid extracts (122.33 ± 1.54 GAE /L vs. 19.5 ± 0.7 GAE /L for SC CaM and 80.94 ± 0.33 GAE /L vs. 9 ± 1.41 GAE /L for βC CaM). Moreover, when assessing the antioxidant activity using the DPPH test, the liquid fractions of milk (without solvent extraction) demonstrate varying levels. Specifically, acid whey and β-casein fractions of camel milk exhibit higher RSA values (15.58 ± 0.5% and 16.03 ± 0.33%, respectively), while sweet whey and sodium caseinate fractions of cow milk show similar trends (17.45 ± 1.21% and 13.56 ± 0.93%, respectively). Notably, the acidic extracts of all cow and camel milk fractions demonstrate robust antioxidant activity, ranging between 95.71 ± 0.64% and 96.68 ± 0.8%.

**TABLE 5:** Phenolic and antioxidants activities of camel and cow milk fractions.

	DPPH-RSA (%)			TPC (mg GAE/L)	
	**Raw**	**Water/ethanol extract**	**HCl/ethanol extracts**	**Water/ethanol extract**	**HCl/ethanol extract**
**Camel milk**					
CaM	9.50 ± 0.58^e^	2.5 ± 0.12^f^	96.68 ± 0.8^a^	55.73 ± 0.7^f,g^	46 ± 6.4^a^
S CaM	7.54 ± 0.14^d^	9.31 ± 0.9^e^	95.71 ± 0.64^a^	32.10 ± 0.64^h^	40.83 ± 8.5^a^
SW CaM	12.00 ± 0.3^c^	49.16 ± 0.6^b^	96.61 ± 0.69^a^	40.30 ± 0.71^h^	22.5 ± 1.94^b,c^
AW CaM	15.58 ± 0.5^a^	53.1 ± 2.3^a^	95.85 ± 0.14^a^	39.07 ± 0.82^h^	18.5 ± 3.5^c^
SC CaM	13.37 ± 1.03^b^	22.38 ± 0.34^d^	95.98 ± 0.97^a^	122.33 ± 1.54^b^	19.5 ± 0.7^c^
β C CaM	16.03 ± 0.33^a^	36.6 ± 2.2^c^	96.35 ± 0.08^a^	80.94 ± 0.33^c^	9 ± 1.41^d^
**Cow milk**					
CoM	8.50 ± 0.30^d,e,f^	2.2 ± 0.12^i^	96.17 ± 0.08^a^	63.00 ± 4.24^d,e^	34.5 ± 6.6^b^
S CoM	7.82 ± 0.43^f^	8.65 ± 0.23^f,g^	96.12 ± 0.3^a^	56.00 ± 1.14^f^	23.5 ± 2.8^b,c^
SW CoM	17.45 ± 1.21^a^	3.62 ± 0.12^h^	95.94 ± 0.42^a^	57.50 ± 6.36^d,e,f^	25.5 ± 2.9^b,c^
AW CoM	12.95 ± 0.51^c,d,e^	13.62 ± 0.34^e^	96.08 ± 0.21^a^	75.00 ± 1.41^d^	9.0 ± 2.8^d^
SC CoM	13.56 ± 0.93^b,c^	2.61 ± 0.25^h,i^	95.85 ± 0.37^a^	127.5 ± 0.71^a^	33.5 ± 3.2^b^
β C CoM	10.43 ± 0.36^e,f^	23.9 ± 0.8^d^	96.4 ± 0.5^a^	26.42 ± 0.94^i^	3.5 ± 1.1^d^

The results are presented as the mean ± standard error. In each row, different letters mean significant differences between average values (*n* = 3; *p* < 0.05). RSA, radical scavenging activity (DPPH assay); TPC, total phenol content; CoM, cow milk; S CoM, skimmed cow milk; AW CoM, acid whey from cow milk; SW CoM, sweet whey from cow milk; SC CoM, sodium caseinate from cow milk; βC CoM, β casein from cow milk; W CaM, whole camel milk; S CaM, skimmed camel milk; AW CaM, acid whey from camel milk; SW CaM, sweet whey from camel milk; SC CaM, sodium caseinate, from camel milk; βC CaM, β casein from camel milk. In each column, different letters mean significant differences between average values (*p* < 0.05).

### 3.4 Antidiabetic activity and its correlation to antioxidants and total essential amino-acids

Table 6 shows the results of α-amylase inhibition as an indicator of *in vitro* antidiabetic activity of cow and camel milk, measured directly on raw milk, protein fractions and on both ethanol/water and HCl /ethanol extracts. The results reveal considerable variability in the proportion of α-amylase inhibition among fractions (skim milk, whey and casein fractions), extraction techniques, and the milk variety (cow or camel) (cow or camel). Notably, the raw whey fractions from both camel and cow milk (SW CaM, AW CoM, SW CaM, and AW CoM) display the highest values, with percentages of 97.43 ± 1.51%, 93.89 ± 1.08%, 87.75 ± 0.57%, and 91.94 ± 6.43%, respectively. When examining the acidic extracts, the percentages of α-amylase inhibition exceed 96%. In contrast, the highest antidiabetic activity for the ethanolic extracts was found for β-casein of cow milk with a percentage of 65.22 ± 0.58%, followed by sodium caseinate of camel milk and sodium caseinate of cow milk (56.98 ± 0.12% and 53.59 ± 0.52%, respectively).

**TABLE 6:** Antidiabetic activity using α-amylase inhibition percentage, I_α_ (%).

Sample	Protein fraction	HCl/ethanol extract	Water/ethanol extracts
**Camel milk**			
CaM	73.59 ± 2.46^e,f^	99.43 ± 0.23^a^	ND
S CaM	76.41 ± 0.66^e^	97.06 ± 0.17^b,c^	ND
SW CaM	97.43 ± 1.51^a^	96.41 ± 0.12^c^	ND
AW CaM	93.89 ± 1.08^b^	96.24 ± 0.12^c^	ND
SC CaM	19.12 ± 1.43^i^	98.82 ± 0.14^a^	56.98 ± 0.12^a^
β C CaM	25.04 ± 2.51^h^	98.86 ± 0.46^a^	13.06 ± 0.23^c^
**Cow milk**			
CoM	30.56 ± 0.87^h^	96.82 ± 0.06^c^	ND
S CoM	4.41 ± 0.16^j,k^	98.69 ± 0.01^a,b^	ND
SW CoM	87.75 ± 0.57^d^	98.78 ± 0.01^a^	ND
AW CoM	91.94 ± 6.43^c^	99.06 ± 0.32^a^	ND
SC CoM	51.17 ± 1.05^g^	99.80 ± 0.09^a^	53.59 ± 0.52^b^
β C CoM	5.27 ± 1.05^j^	99.51 ± 0.29^a^	65.22 ± 0.58^a^

The results are presented as the mean ± standard error. In each row, different letters mean significant differences between average values (*n* = 3; *p* < 0.05). ND, not detected; CoM, cow milk; S CoM, skimmed cow milk; AW CoM, acid whey from cow milk; SW CoM, sweet whey from cow milk; SC CoM, sodium caseinate, from cow milk; βC CoM, β casein from cow milk; W CaM, whole camel milk; S CaM, skimmed camel milk; AW CaM, acid whey from camel milk; SW CaM, sweet whey from camel milk; SC CaM, sodium caseinate, from camel milk; βC CaM: β casein from camel milk. In each column, different letters mean significant differences between average values (*p* < 0.05).

**FIGURE 3:**
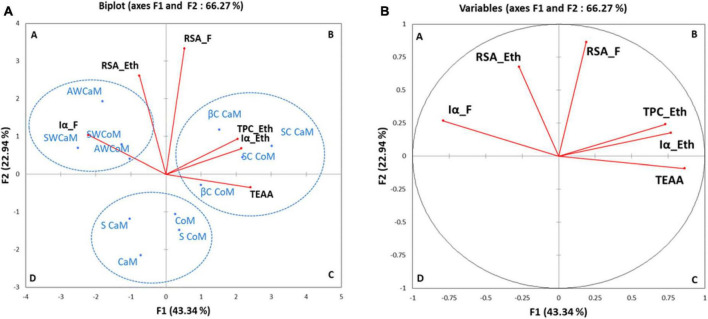
Principal component analysis (PCA) biplot of objects and component loads for grouping of descriptors for bioactive contents (Iα_F, RSA_Eth, RSA_F, TPC_Eth, Iα_Eth and TEAA) **(A)** and groups of milk fractions samples **(B)**. CoM, whole cow milk; S CoM, skim cow milk; AWCoM, acid whey from cow milk; SW CoM, Sweet whey from cow milk; SC CoM, sodium caseinate, from cow milk; βC CoM, β casein from cow milk; CaM, whole camel milk; S CaM, skim camel milk; AW CaM, acid whey from camel milk; SW CaM, sweet whey from camel milk; SC CaM, sodium caseinate, from camel milk; βC CaM, β casein from camel milk. Iα_F, α-amylase inhibition of protein fractions; TEAA, Total essential amino acids; RSA_Eth, Radical scavenging activity of ethanolic extracts; RSA_F, Radical scavenging activity of raw fractions; TPC_Eth, Total phenolic content of ethanolic extracts; Iα_Eth, α-amylase inhibition of ethanolic extracts.

To better understand correlation between antioxidant (TPC, DPPH-RSA), total essential amino-acids (TEAA) with *in vitro* α amylase inhibition, the PCA was performed. Figure 3 showed the PCA biplot of TPC, TEAA, RSA, and Iα of milk as well as the respective whey and casein fractions and their ethanolic extracts. The plot distinguishes four quadrants denoted as A, B, C, and D, each with its own set of elements. The plot revealed that PCA accounted 66.27% of data variation across the initial two elements. Specifically, PC1 accounted for 43.34% of the variance, while PC2 contributed an additional 22.94% to the overall variance. The first dimension is favorably impacted by the RSA of raw fractions (RSA_F) (0.189), TPC of ethanolic extracts (TPC_Eth) (0.730), α-amylase inhibition in ethanolic extracts (Iα_Eth) (0.768), and TEAA (0.862). In quadrant A, the initial group comprises whey fractions from both camel and cow milk (AW CoM, AW CaM, SW CoM, and SW CaM), exhibiting a positive correlation with radical scavenging activity and α-amylase inhibition. A second group composed of casein fractions (SC and βC) of both cow and camel milk having a positive correlation with TEAA, TPC, Iα of ethanolic extracts was observed in quadrant B. Skim and whole camel and cow milk (CaM, S CaM, CoM and S CoM) form a third group apart (quadrants C and D) with a negative correlation with radical scavenging and α amylase inhibition (Iα).

## 4 Discussion

The current study focused on investigating the protein and amino acid profiles, alongside the antioxidant and antidiabetic properties, of milk fractions derived from both camel and cow milk through fractionation processes. The findings highlighted significant variations in protein composition between the two milk types. Specifically, β-casein dominated the protein content in sodium caseinate, β-lactoglobulin prevailed in cow whey fractions, and α-lactalbumin was the prominent protein in camel whey fractions. Our milk protein profiles are consistent with previous research (6, 25), which also observed similar patterns in milk protein profiles. The α-lactalbumin stands as the principal dominant soluble protein in camel milk fractions, while β-lactoglobulin is absent. Moreover, β-casein emerged as a principal insoluble protein in both camel and cow milk.

Furthermore, our analysis of amino acid content revealed significant patterns. In skim milk and sweet whey milk from both camel and cow sources, leucine, lysine, and proline emerged as the primary amino acids, accounting for a substantial portion (ranging from 1 to 5.2%) of the amino acid composition. In contrast, acid whey, casein fractions (both sodium caseinate and β-casein) produced from both camel and cow milk exhibited an elevated levels of histidine, leucine, lysine, and proline (ranging from 1.12 to 6.62%). This result aligns with the conclusions made by Rafiq et al. (26) which stated that casein fractions from several milk species, including camel and cow milk, had the highest amount of leucine and the second-elevated concentration of lysine. Similarly, Salmen et al. (27) reported the following relative abundance of amino acids in casein of camel and cow milk: lysine, 7.5 ± 0.35 and 9.78 ± 0.19; threonine, 4.05 ± 0.05 and 4.00 ± 0.19; leucine, 7.58 ± 0.18 and 8.24 ± 0.2; valine, 5.63 ± 0.15 and 6.17 ± 0.17; and isoleucine, 4.43 ± 0.16 and 4.64 ± 0.18 (g amino acid/100 g protein), respectively. Leucine has a vital function in initiating the production of protein in muscles, regulating protein metabolism, and assisting in the reversible phosphorylation of proteins required for mRNA connecting to the 40S ribosomal subunit (28). In contrast, all fractions from cow and camel milk contained equivalent levels of asparagine and glutamine. Through the conversion of intracellular glutathione, sulfur-containing amino acids (such methionine) enhance immunological function and act as antioxidants (29). These proteins have garnered substantial interest for their potential to fortify host defenses through specific dietary interventions. In another hand, our results are consistent with those of Eyassu (8), who reported a significant concentration of essential amino acids in camel milk proteins. Notably, previous reports didn’t provide complete amino acid profiles for both camel sweet and acid whey, sodium caseinate, β-casein, and corresponding skim milk. These data hold valuable impacts on the establishment of innovative strategies for milk whey utilization, innovative food formulations, and tailored dietary applications.

Camel and cow milk fractions were evaluated for phenolic content and radical scavenging potential. It was found that acid extraction of milk enables the production of milk fractions that have lower TPC than the equivalent skim milk, while TPC contents of water/ethanol extracts show higher values of TPC than those obtained in HCl/ethanol extracts with a significant variability between fractions. While many authors have reported on the antioxidant activities of various milk forms, including liquid milk (16, 30), fermented milk (15), milk hydrolysates (16, 31), and heat-treated milk (30), there is a relative scarcity of reports focusing on sweet, acid whey, or casein fractions (17, 25). Moreover, within the same milk species, the outcomes fluctuated depending on the specific antioxidant assay employed (DPPH assay, ABTS assay, iron-binding capacity) and the kind of product studied (unprocessed milk, processed dairy products, or milk extracts). The differences in antioxidant activity observed can be ascribed to the distinct antioxidant profiles of milk fractions. Notably, the antioxidant activity (% of inhibition) varies depending on the type of extract. In the case of acidic extracts, it is primarily the phenolic compounds that contribute to the observed antioxidant activity. In the case of ethanolic extracts, both phenolic compounds and proteins contribute to the whole antioxidant activity. These differences suggest the possibility of antagonist and synergistic effects among various compounds present in milk fractions. Furthermore, these findings may provide an explanation for the lower DPPH-RSA values observed in whole and skim milk when compared to isolated protein fractions. Notably, ethanolic extracts from whey fractions (acid and sweet whey) of camel milk, as well as β-casein fractions from both camel and cow milk, displayed significantly higher DPPH-RSA values, ranging from 23.9 to 53%, in comparison to other fractions. This heightened percentage of inhibition maybe related to the enriched content of α-lactalbumin within these fractions, as confirmed by HPLC protein profiles (AW CoM∼ 34% α-lactalbumin vs. AW CaM∼61.5% α-lactalbumin, SW CoM ∼29.5% α-lactalbumin vs. SW CaM ∼ 49% α-lactalbumin). The presence of α-lactalbumin in these fractions may interact with phenolic compounds, contributing to the overall DPPH-RSA. Many authors (32, 33) discovered a variety of peptides derived from α-lactalbumin and β-lactoglobulin that were located in various key regions and showcasing notable antioxidant attributes. Furthermore, it is noteworthy that whey proteins possess the capacity to enhance the activity of glutathione peroxidase, a pivotal water-soluble antioxidant system within milk (34). Several biologically active substances, such as vitamins (E and C), retinol, β-carotene, enzymes (superoxide dismutase, catalase, and glutathione peroxidase), have also demonstrated significant antioxidant potential following deproteinization (35). Additionally, milk boasts a substantial concentration of equol, a phenolic metabolite derived from daidzein, which has been shown to possess antioxidant activity (36). Moreover, our findings indicate that camel-casein (SC CaM and βC CaM) and their ethanolic extracts exhibit significantly elevated RSA-DPPH levels in comparison to cow casein (SC CoM and βC CoM). The capacity of proteins to function as antioxidants can be attributed to various factors, including their amino acid composition, structural configuration, hydrophobicity index, and accessibility (37). When examining the amino acid profiles, it becomes evident that camel-casein boasts a higher abundance of antioxidant amino acids, including leucine, lysine, isoleucine, and proline, in comparison to cow-casein (Tables 3, 4).

Acidic extracts displayed remarkable α-amylase inhibition rates, exceeding 96%. It’s important to note that the acidic extraction process results in the depletion of proteins. Consequently, the α-amylase inhibitory activity observed in the acidic extracts is attributed to the bioactive compounds that remain within the deproteinized milk matrix. These compounds include vitamin C and phenolic compounds. Conversely, ethanolic extracts exhibited varying levels of antidiabetic activity, with the highest activity detected in cow milk’s β-casein (65.22 ± 0.58%), followed by sodium caseinate from the two types of milk (56.98 ± 0.12% and 53.59 ± 0.52%, respectively). The variations of radical scavenging activities and antidiabetic activities assessed in ethanolic and acidic extracts are not only attributed to differences between sample composition but they also depend on the used solvents. It was reported that the use of DPPH in acid reaction mixtures has the potential to produce misleading positive outcomes. Indeed, the presence of acid in the solvent may influence the ionization equilibrium (38, 39). The acidic condition may result in a false positive reduction in the assay’s color, leading to an overestimation of the antioxidant activity.

The antidiabetic effect of milk fractions compared to their extracts has not been previously investigated. These findings suggest that the *in vitro* antidiabetic properties of milk and its protein fractions may be linked to their ability to inhibit enzymes involved in glucose metabolism, as indicated by Ayyash et al. (15). Milk contains a high concentration of potent antidiabetic compounds, including antioxidants like phenolic compounds and vitamins, as well as active peptides (35). Numerous research studies have explored the potential antidiabetic properties of milk, yielding variable outcomes based on the type of antidiabetic assays utilized and the kind of milk under investigation. These assessments covered various enzymatic actions, including inhibiting α-amylase, α-glucosidase, DPP-IV, and lipase. The complexity of the results was further influenced by the type of milk examined, whether it was raw milk, fermented milk, or milk subjected to hydrolysis. For instance, it was found that some components derived from cow milk, namely whey protein isolate, α-lactalbumin, and β-lactoglobulin, exhibited inhibitory effects on both DPP-IV and α-glucosidase (40). On the other hand, other research has highlighted a potential connection between diabetes and certain components and proteins found in milk produced by camels, including lactoferrin (41). Furthermore, recent investigations have provided evidence for the existence of biologically active molecules, particularly proteins and peptides in camel milk. These bioactive components appear to be capable of inhibiting three essential metabolic enzymes associated with diabetes: porcine pancreatic α-amylase (PPA), dipeptidyl peptidase-IV (DPP-IV), and pancreatic lipase (PPL) (42). This discovery highlighted milk fractions as a promising source of antidiabetic agents with the potential to offer new avenues for diabetes management.

The application of principal component analysis (PCA) in our investigation allowed us to illustrate the relationships between various factors, including antioxidants (TPC and DPPH-RSA), total essential amino acids, and *in vitro* α-amylase inhibition. The analysis revealed a positive correlation between TPC, RSA-DPPH, and α-amylase inhibition. This discovery emphasizes the significant impact of the phenolic and amino acid profiles in milk fractions, which contribute to their antioxidant and antidiabetic properties. This underscores the vital role played by these bioactive components in enhancing the health-promoting attributes of milk fractions, particularly their antioxidant and antidiabetic properties.

## 5 Conclusion

The proteins and amino-acids profiles, antioxidant and antidiabetic properties of camel and cow milk fractions produced by enzymatic, or acid coagulation were investigated. The results suggested that fractionation of milk allows to obtain fractions with different protein and amino-acids compositions endowed with interesting distinctive antioxidant and antidiabetic activities. Positive correlations were observed between radical scavenging activities and α amylase inhibition of whey fraction. Whereas casein fraction showed positive correlation between total essential amino acids, total phenols and α amylase inhibition. Whey fractions of camel milk exhibited higher antioxidant and antidiabetic activities than corresponding skim milk. Acid and sweet whey fractions of camel milk seemed to be the most interesting for further exploration of *in vivo* antioxidant and antidiabetic activities and are promising milk derivatives for specific nutritional diet and/or functional food formulation.

## Data availability statement

The raw data supporting the conclusions of this article will be made available by the authors, without undue reservation.

## Author contributions

NH: Data curation, Formal analysis, Investigation, Methodology, Software, Writing – original draft. AZ: Methodology, Validation, Writing – review and editing. NR: Formal analysis, Methodology, Validation, Investigation, Writing – review and editing. MB: Investigation, Methodology, Validation, Writing – review and editing. NM’h: Investigation, Methodology, Validation, Writing – review and editing. AS: Investigation, Methodology, Resources, Writing – review and editing. MA: Conceptualization, Resources, Supervision, Writing – review and editing. NB: Conceptualization, Funding acquisition, Methodology, Project administration, Resources, Supervision, Validation, Visualization, Writing – review and editing.
